# Altered regional homogeneity and functional brain networks in Type 2 diabetes with and without mild cognitive impairment

**DOI:** 10.1038/s41598-020-76495-3

**Published:** 2020-12-04

**Authors:** Ying Xiong, Xiaodan Chen, Xu Zhao, Yang Fan, Qiang Zhang, Wenzhen Zhu

**Affiliations:** 1 Department of Radiology, Tongji Hospital, Tongji Medical College, Huazhong University of Science and Technology, 1095 Jiefang Ave, Wuhan, 430030 China; 2 State Key Laboratory of Cognitive Neuroscience and Learning and IDG/McGovern Institute for Brain Research, Beijing Normal University, Beijing, China; 3 GE Healthcare, Beijing, China; 4 Department of Neurology, Tongji Hospital, Tongji Medical College, Huazhong University of Science and Technology, 1095 Jiefang Ave, Wuhan, 430030 China

**Keywords:** Neuroscience, Medical research

## Abstract

Patients with Type-2 Diabetes Mellitus (T2DM) have a considerably higher risk of developing mild cognitive impairment (MCI) and dementia. The initial symptoms are very insidious at onset. We investigated the alterations in spontaneous brain activity and network connectivity through regional homogeneity (ReHo) and graph theoretical network analyses, respectively, of resting-state functional Magnetic Resonance Imaging (rs-fMRI) in T2DM patients with and without MCI, so as to facilitate early diagnose. Twenty-five T2DM patients with MCI (DM-MCI), 25 T2DM patients with normal cognition (DM-NC), 27 healthy controls were enrolled. Whole-brain ReHo values were calculated and topological properties of functional networks were analyzed. The DM-MCI group exhibited decreased ReHo in the left inferior/middle occipital gyrus and right inferior temporal gyrus, and increased ReHo in frontal gyrus compared to the DM-NCs. Significant correlations were found between ReHo values and clinical measurements. The DM-MCI group illustrated greater clustering coefficient/local efficiency and altered nodal characteristics (efficiency, degree and betweenness), which increased in certain occipital, temporal and parietal regions but decreased in the right inferior temporal gyrus, compared to the DM-NCs. The altered ReHo and impaired network organization may underlie the impaired cognitive functions in T2DM and suggesting a compensation mechanism. These rs-fMRI measures have the potential as biomarkers of disease progression in diabetic encephalopathy.

## Introduction

The global prevalence of Type-2 Diabetes Mellitus (T2DM) has been rapidly increasing. The International Diabetes Federation has released new estimates on the prevalence of diabetes worldwide, indicating that 1 in 11 adults are currently living with diabetes, which is 10 million more individuals than reported in 2015^1^. Patients with T2DM demonstrate an increased risk of Alzheimer’s disease (AD) and cognitive impairment, which commonly manifests as declining memory, information processing speed, and learning and executive functions^2,3^. However, the pathophysiological mechanism of T2DM-induced cognitive impairment has not yet been elucidated. Previous studies suggested that T2DM and AD may share several patterns of brain pathogenesis, such as impaired insulin sensitivity and signaling, cerebral amyloid beta aggregation and tau hyperphosphorylation^4^.

The pathophysiology of cognitive decline in the diabetic brain has aroused much interest owing to its high incidence. Neuroimaging techniques can provide important clues regarding brain structure and function for understanding the neurological involvement in T2DM patients. Morphological atrophy was considered related and observed in white and gray matter, including hippocampal structures^5,6^. Moreover, white matter lesions^7^, reduced white matter integrity^8,9^, decreased density of axons/dendrites^10^, and altered cerebral metabolism^11^ were detected and associated with cognitive dysfunction.

Besides structural and metabolic information, neural activity is a sensitive functional measurement that can be acutely altered together with structural measures of brain lesions^12^. Neural activity may provide clues to track the early effects of diabetic causative factors. Resting-state functional Magnetic Resonance Imaging (rs-fMRI) can noninvasively detect spontaneous neural activity at baseline and be used to further investigate the local and global properties of functional brain networks. Currently, rs-fMRI is commonly used to study cognitive function in neuropsychological disorders^13^. Regional homogeneity (ReHo) is one of the main metrics used to assess the local characteristics of rs-fMRI signals. It has been used to analyze the synchronization of a given voxel with its neighboring voxels^14^. ReHo values in T2DM patients were reported to decrease in the occipital lobe, postcentral gyrus and fusiform gyrus, and increase in the medial frontal gyrus and anterior cingulate gyrus^15,16^, indicating altered local neuronal synchronization in these regions. Furthermore, The brain is organized into segregated complex systems with different functional areas that are specialized for processing distinct information. Information exchanges between interconnected brain regions are thought to be the biological basis for human cognitive processes^17^. Graph theory-based network analysis is an effective method to investigate the topological organizations of brain functional networks^18^. This analysis has been instrumental in understanding the underlying mechanisms of many neuropsychlogical disorders such as AD, epilepsy and schizophrenia^18^. Graph theory-based network analysis demonstrated altered topological organizations of the brain network in a group of T2DM patients, including those with normal cognition and impaired cognition^19–21^. As effective indicators reflecting the intrinsic organization of the resting brain, ReHo and network connectivity approaches have been conjunctively applied in studies of complex functional activity in AD^22^.

Previous rs-fMRI studies regarding the diabetic brain have focused primarily on differences between T2DM patients and healthy controls, which have demonstrated altered patterns of brain activity associated with cognitive abnormalities in T2DM patients. However, whether these changes are the result of early stage dementia or T2DM factors, such as neurodegeneration caused by advanced glycation end products (AGEs) or toxic effects from high blood glucose^23^, needs to be further investigated. Moreover, it is important to note that some patients with diabetes progress to mild cognitive impairments (MCI) or dementia rapidly, while other patients only demonstrate a similar range compared to normal cognitive decline with aging. We hypothesized that altered ReHo values and topological organizations would be detected within specific brain regions. Applying voxel-based ReHo analysis of brain activity and graph theory analysis of functional connectivity in a same dataset, we aimed to investigate the possible changes of both local and global functional brain activities between T2DM patients with MCI and without MCI.

## Material and methods

### Participants

A cross-sectional study design was conducted in this research. With the approval of the Institutional Review Board of Tongji Medical College, Huazhong University of Science and Technology, 54 participants (51–72 years of age, 30 female) with confirmed T2DM were recruited from the endocrinology clinical service. Twenty-seven of the patients had mild cognitive impairment (DM-MCI group), while 27 patients had normal cognition (DM-NC group). A battery of neurocognitive tests was performed to diagnose MCI and assess their cognitive functions, as detailed in the next subsection. Detailed information regarding hypoglycemic agent application, family history, clinical complaints and complications was recorded. Clinical examinations, including measurements of blood biochemistry, lipids, cholesterol, plasma glucose, glycosylated hemoglobinA1c (HbA1c), and body mass index (BMI), were carefully performed by specialists. The diagnosis of T2DM was based on standard criteria from the American Diabetes Association^24^. Twenty-seven euglycemic subjects (51 to 73 years of age, 15 female, fasting glucose level < 7.0 mmol/L, HbA1c < 6.0%, without diabetes family history) were also enrolled as healthy controls (HC group). The exclusion criteria included the following: (a) lesions in the brain, such as tumors, cerebral infarction, hemorrhage, or vascular malformation; (b) a history of stroke, epilepsy, head trauma, or brain surgery; (c) systemic organic disease or a history of tumors; (d) other types of diabetes; and (e) contraindication to MRI examination, such as the presence of metallic implants or claustrophobia. All participants were right-handed and matched by age, gender, and education by group totals.

### Neuropsychological assessments

All subjects underwent comprehensive physical, neurological, and neuropsychological assessments, which included the Mini-Mental State Examination (MMSE), Montreal Cognitive Assessment (MoCA), Hachiski test, Activity of Daily Living (ADL) test, and Auditory Verbal Learning test (AVLT). MMSE and MoCA tests were performed at 2-week interval again for reliability.The inclusion criteria for DM-MCI group, same as our previous study^8,10^, required that patients demonstrated the following: (a) complaints of memory decline, which occurred after clinical T2DM diagnosis; (b) both MoCA and MMSE scores ≤ 27; and (c) absence of any other physical or mental disorders that can lead to cognitive impairment. The Hachiski test was used to exclude vascular dementia (N = 0). Demographics, clinical data and cognitive assessment results were compared among the three groups using a one-way Analysis of Variance test (ANOVA), a Student’s *t*-test and a Pearson chi-square test with SPSS software (SPSS Inc., Chicago, IL, USA).

### MRI image acquisition

Images were acquired on a 3-T MRI scanner (Discovery MR750, GE Healthcare, Waukesha, WI, USA) using a commercial 32-channel head coil. The subjects were instructed to close their eyes but stay awake during the scanning (monitored by MR technicians from a screen outside). High-resolution anatomical images were obtained with a sagittal T1-3D brain volume imaging sequence (repetition time/echo time/inversion time = 8.2/3.2/450 ms, flip angle = 12°, section thickness = 1 mm, matrix size = 256 × 256 × 160, field of view = 25.6 × 25.6 cm^2^, and NEX = 1) for radiological evaluation and identifying lesions specified in the exclusion criteria. Functional images were obtained axially using a gradient-echo echo planar imaging sequence with the following parameters: repetition time = 2000 ms, echo time = 35.0 ms, field of view = 24.0 × 24.0 cm^2^, matrix size = 64 × 64, slice thickness = 4.0 mm without spacing, acquisition bandwidth = 250 kHz, and flip angle = 90°. In total, 240 volumes were acquired interleaved head-to-foot, and the scan time was 8 min. Scan planes were axially positioned and covered the whole brain, including the brain stem and cerebellum.

### Data preprocessing and ReHo analysis

Functional image preprocessing and ReHo calculation were conducted with the Data Processing & Analysis of Brain Imaging toolkit (DPABI v3.0, www.nitrc.org/projects)^25^ and SPM12 (www.fil.ion.ucl.ac.uk/spm) software. The first 10 volumes were removed, taking into account the magnetization equilibrium. The remaining images were processed with the following steps. First, fMRI images were corrected for slice timing and realigned to the mean image to correct for head movement using Friston 24-parameter motion^26^ correction and framewise displacement value calculation (the bad time points could be flagged by any volume with framewise displacement > 0.2 mm)^25^. Two participant from the each DM group was excluded from further data analysis because of excessive head motion (> 2 mm of displacement or > 2° of rotation). The realigned images were spatially normalized to a standard template in the Montreal Neurological Institute (MNI) space and resampled to 3 × 3 × 3 mm isotropic voxel size (T1 structural image were used in this process). Then, detrending and nuisance regression procedures were performed to remove linear trends and nuisance signals from the image time series, and the data were filtered at the 0.01–0.1 Hz band to remove the effects of low-frequency drift and high-frequency noise. The ReHo calculation was performed on the preprocessed images. Individual ReHo maps were generated by calculating the Kendall coefficient concordance to measure the similarity of the time series of a given voxel and its 26 nearest neighbors in a voxel-wise way^14^. Finally, a z-transformation was conducted on the individual ReHo maps to generate normally distributed zReHo maps.

### Within-Group and between-group statistical analysis

One-sample *t*-tests were performed on individual zReHo maps for each group using Statistical Analysis in DPABI^25^. A statistical significance threshold was set at *p* < 0.001 and a false discovery rate (FDR) correction was applied for multiple comparisons with *p* < 0.005. Group comparisons of ReHo values were performed (within a Gray-Matter mask) with one-way Analysis of Covariance (ANCOVA) with age, gender, and education level as covariates, and post-hoc pairwise comparisons were performed by a general linear regression model if ANCOVA yielded significant results. The statistical threshold was set at *p* < 0.01 and a minimum cluster size of 80 voxels, which corresponded to a corrected p < 0.01 (AlphaSim correction; http://afni.nih.gov/afni/docpdf/AlphaSim.pdf).

To investigate the relationship between ReHo values, cognitive performance, and diabetes-related parameters (fasting plasma glucose/HbA1c levels and disease duration), Pearson^’^s correlation analyses were performed in a voxel-wise manner adjusted by age, gender, and education level covariates using the DPABI software. A statistical threshold was set at *p* < 0.01 (AlphaSim correction) to explore the most significant correlations among MR voxels.

### Functional network analysis

The preprocessed rs-fMRI data were segmented into 90 regions (45 in each hemisphere) using the anatomically labeled (AAL) template reported by Tzourio-Mazoyer^27^. Each region represented one node of the brain network. A few sparsity thresholds ranging from 0.1–0.34 with an interval of 0.01 were applied as suggested^21,28^. Graph theoretical analysis was carried out using GRETNA software^29^. For brain networks at each sparsity threshold, we calculated global and regional network parameters, which involved (1) small-world parameters (normalized characteristic path length λ, normalized clustering coefficient γ, small-worldness σ), clustering coefficient Cp, and characteristic path length Lp; (2) network efficiency measures: global efficiency Eg and local efficiency Eloc, and (3) nodal parameters (efficiency, degree and betweenness). Then, the area under the curve (AUC) for each network metric was calculated, which was sensitive at detecting topological alterations and provided a summarized scalar for the topological characterization of brain networks^20,21^. The AUCs were calculated for each parameter over the entire sparsity range in this study (0.1 ≤ Sp ≤ 0.34). The network analyses were visualized using BrainNet Viewer^30^ software. A one-way ANOVA was performed on the AUC of all network metrics, and the statistically significant level was set at *p* < 0.05. For nodal parameters, an FDR correction was applied for multiple comparisons.

### Ethical approval

The current study was approved by the Research Ethics Committee of the Tongji Medical College, Huazhong University of Science and Technology. Informed consent was obtained from all individual participants included in the study. All methods were carried out in accordance with relevant guidelines and regulations (Declaration of Helsinki).

## Results

### Sample characteristics

The clinical and neuropsychological characteristics of the three groups are summarized in Table 1. No significant differences were observed among the three groups for age, gender, years of education and BMI. Although no significant differences were observed in fasting and postprandial glucose levels, the DM-MCI group exhibited a higher level of glycosylated HbA1c (*p* = 0.014) and a trend towards an increase in disease duration (*p* = 0.073) compared to the DM-NC group. The DM-MCI group had significantly lower MoCA and MMSE scores than both the DM-NC and HC groups and performed worse on the AVLT, indicating a decline in verbal memory.

**Table 1:** Sample characteristics.

Clinical information	DM-MCI group (n = 25)	DM-NC group (n = 25)	Healthy controls (n = 27)	*p*-value^a^
Gender (female: male)	14:11	15:10	15:12	0.940*
Age (years)	62.68 ± 5.65	59.05 ± 6.22	59.08 ± 6.35	0.110^†^
Formal education (years)	11.09 ± 3.50	11.64 ± 3.32	11.20 ± 2.60	0.829^†^
BMI (kg/m^2^)	23.73 ± 2.90	22.63 ± 2.53	24.01 ± 2.04	0.142^†^
Hypertension^b^	5(20)	4(16)	3(11.1)	0.676*
Hyperlipidemia^c^	2(8)	4(16)	2(7.4)	0.534*
Diabetes duration (years)	8.97 ± 7.48	5.69 ± 4.57	–	0.073
Family history^d^	5(20)	7(28)	–	0.508*
Insulin treated	2(8)	0(0)	–	0.149*
Complications (peripheral nerve and vascular lesions; Retinopathy)	4(16)	1(4)	–	0.157*
Fasting glucose (mmol/L)	9.63 ± 2.22	10.30 ± 3.35	5.22 ± 0.64	0.441
Postprandial glucose	13.94 ± 4.74	14.99 ± 4.61	–	0.458
HbA1c (mmol/mol)	66.69 ± 17.46	54.71 ± 14.93	34.81 ± 4.40	0.014
HbA1c (%)	8.26 ± 1.60	7.16 ± 1.36	5.27 ± 0.41	0.014
MMSE	25.29 ± 2.05	28.55 ± 0.96	28.40 ± 1.04	< 0.001
MoCA	25.25 ± 1.19	28.09 ± 0.68	28.80 ± 1.08	< 0.001
AVLT	29.5 ± 8.73	36.11 ± 8.50	–	0.028
Hachiski	1.91 ± 1.02	2.09 ± 1.06	–	0.566
ADL (Barthel index)	99.55 ± 1.47	99.41 ± 2.77	–	0.839

Data are expressed as the mean ± standard deviation or percentage number (%) unless otherwise indicated.

^a^*p*-values labeled with * and ^†^ were obtained using a Pearson Chi-square test (2-sided) and an ANOVA, respectively. All other *p*-values were obtained using a 2-tailed Student’s *t*-test between the DM-MCI and the DM-NC groups.

^b^Hypertension: systolic pressure ranged between 140–159 mmHg or diastolic pressure ranged between 90–99 mmHg. Patients with moderate and severe hypertension were excluded.

^c^Hyperlipidemia was evaluated as cholesterol > 5.7 mmol/L or triglyceride > 1.7 mmol/L.

^d^Family history accounts for immediate family members who had T2DM within three generations.

### ReHo analysis

**Figure 1:**
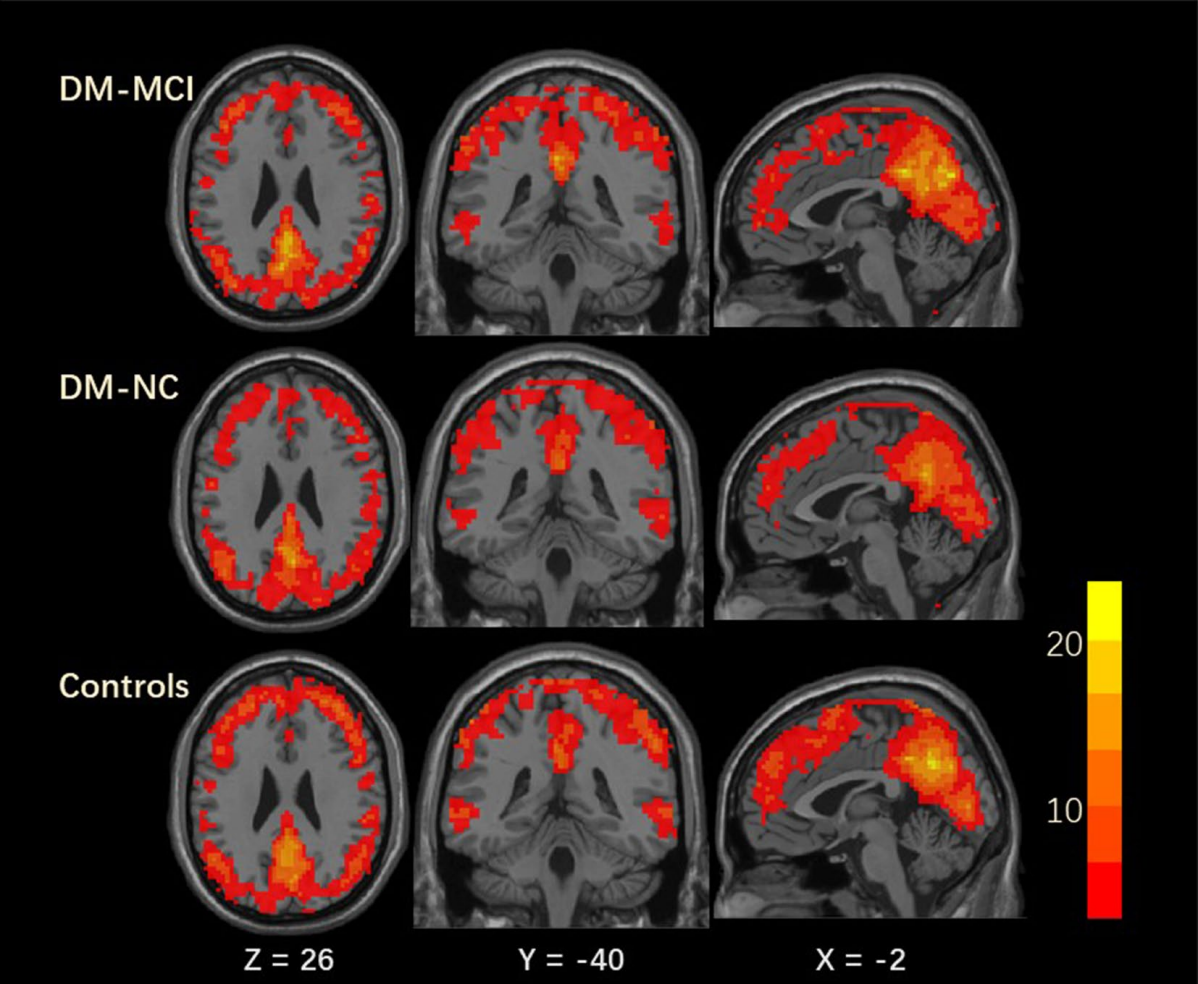
Representative slices of whole brain showing the one-sample *t*-test result of ReHo maps in DM-MCI, DM-NC and HC groups (corrected* p* < 0.005). Within each group, standardized ReHo values in the bilateral frontal/parietal/occipital cortex, the posterior cingulate cortex and precuneus, including main parts of the default-mode network, were significantly higher than the global mean values. The color scale denotes the *t* value.

**Figure 2:**
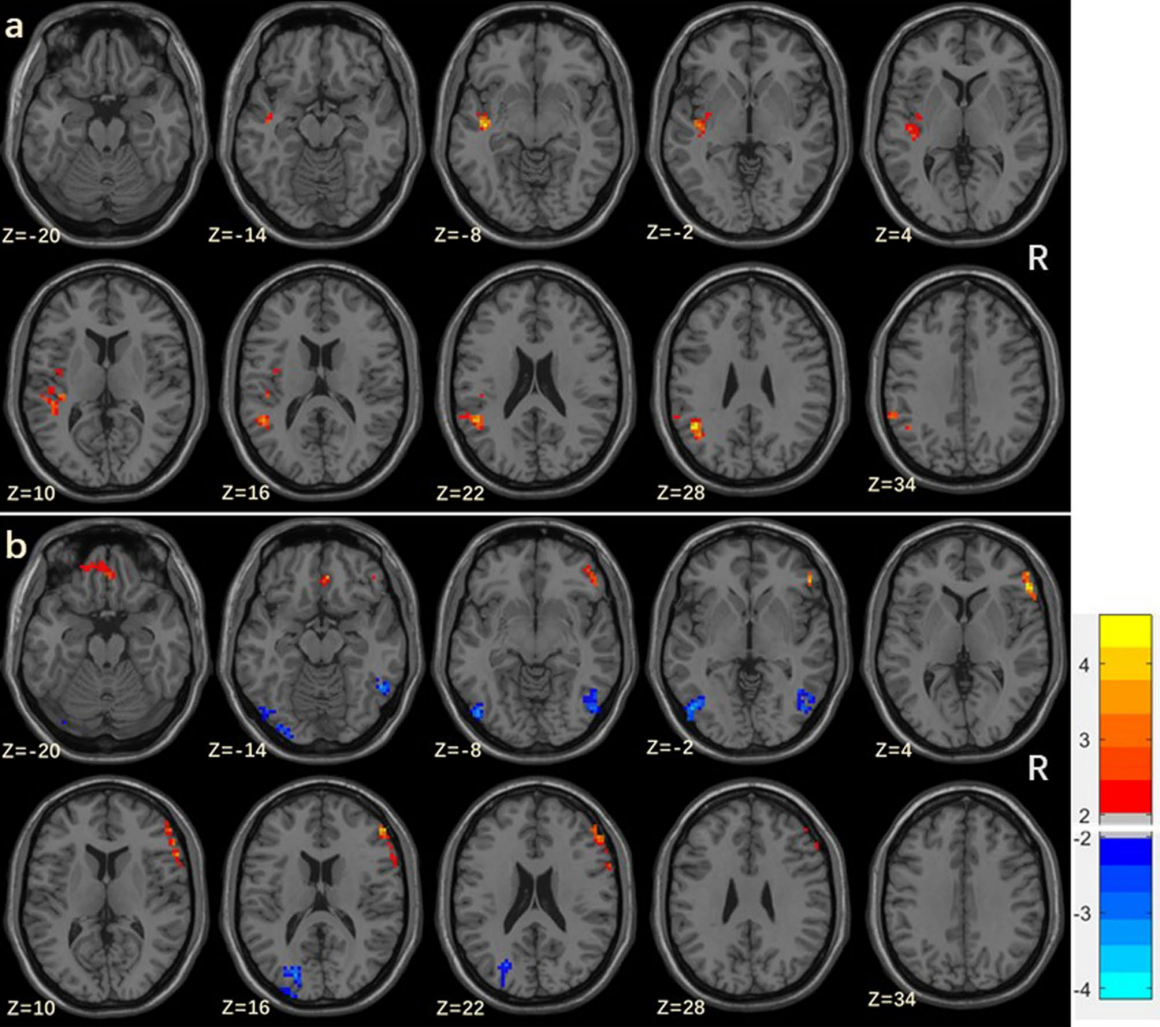
The results showing ReHo differences between DM-NC and HC groups (**a**), and between DM-MCI and DM-NC groups (**b**) (*p* < 0.01, AlphaSim corrected). The color bar in represents the T values. *R* right side.

In each group, zReHo values in the bilateral frontal/parietal/occipital cortex, the posterior cingulate cortex and precuneus, which include main parts of the default-mode network, were significantly higher than the global mean values (Fig. 1). Between-group analysis demonstrated that the DM-NC group exhibited increased ReHo values in the left angular and superior temporal gyrus compared with the HCs. No significantly decreased ReHo values were detected. Furthermore, the DM-MCI group exhibited decreased ReHo values in the left inferior occipital gyrus, the middle occipital gyrus, and the right inferior temporal gyrus compared with the DM-NC group, while increased ReHo values were observed in rectus gyrus and the right inferior frontal gyrus triangular part (Fig. 2 and Table 2).

**Table 2:** Regions showing significant differences on ReHo of subjects’ groups (*p* < 0.01, AlphaSim corrected).

Brain regions (AAL)	BA	Peak MNI coordinates *x y z* (mm)	Peak *t* score	Cluster size
**Increased ReHo in DM-NC than HC**				
Angular_L	39	− 45, − 54, 30	4.655	120
Temporal_Sup_L	48	− 43, − 15, -5	4.631	146
**Increased ReHo in DM-MCI than DM-NC**				
Frontal_Inf_Tri_R	45	54, 30, 3	5.029	167
Recus_R	11	3, 39, − 18	3.508	80
**Decreased ReHo in DM-MCI than DM-NC**				
Occipital_Inf_L	19	− 48, − 72, − 6	− 4.200	105
Occipital_Mid_L	19	− 20, − 82, 16	− 3.640	95
Temporal_Inf_R	37	51, − 54, − 14	− 3.636	115

*AAL* anatomically labeled template, *BA* Brodmann’s area, *MNI* Montreal Neurological Institute.

**Figure 3:**
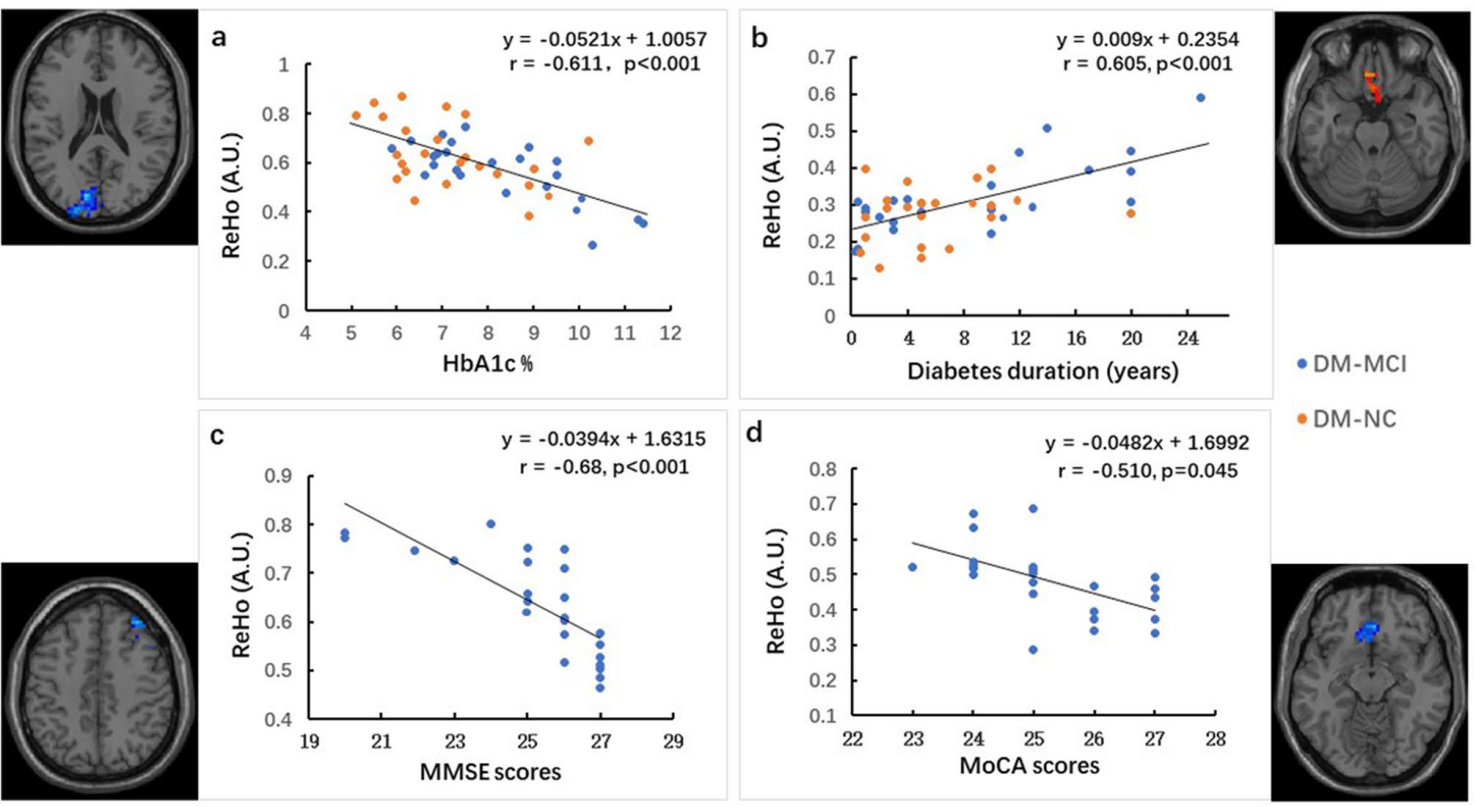
Correlations between the ReHo values and HbA1c (**a**, in the left cuneus) or diabetes duration (**b**, in the rectus gyrus) in all the T2DM subjects, as well as between the ReHo values and the neurocognitive assessments (**c**, in the right middle frontal gyrus and d, in the superior frontal gyrus; the correlation was performed using data from DM-MCI group). Results from the linear regression are indicated in the figure (r: correlation coefficient).

As shown in Fig. 3, the ReHo values were significantly correlated with HbA1c level in the left cuneus (r =  − 0.611, Fig. 3a) and diabetic duration in left rectus gyrus (r = 0.605, Fig. 3b) for all the T2DM subjects. Moreover, the ReHo values correlated with MMSE/MoCA scores in the right middle frontal gyrus (r =  − 0.68, Fig. 3c), the superior frontal gyrus(medial orbital) (r =  − 0.510, Fig. 3d) (*p* < 0.01, AlphaSim corrected).

### Functional network analysis

**Figure 4:**
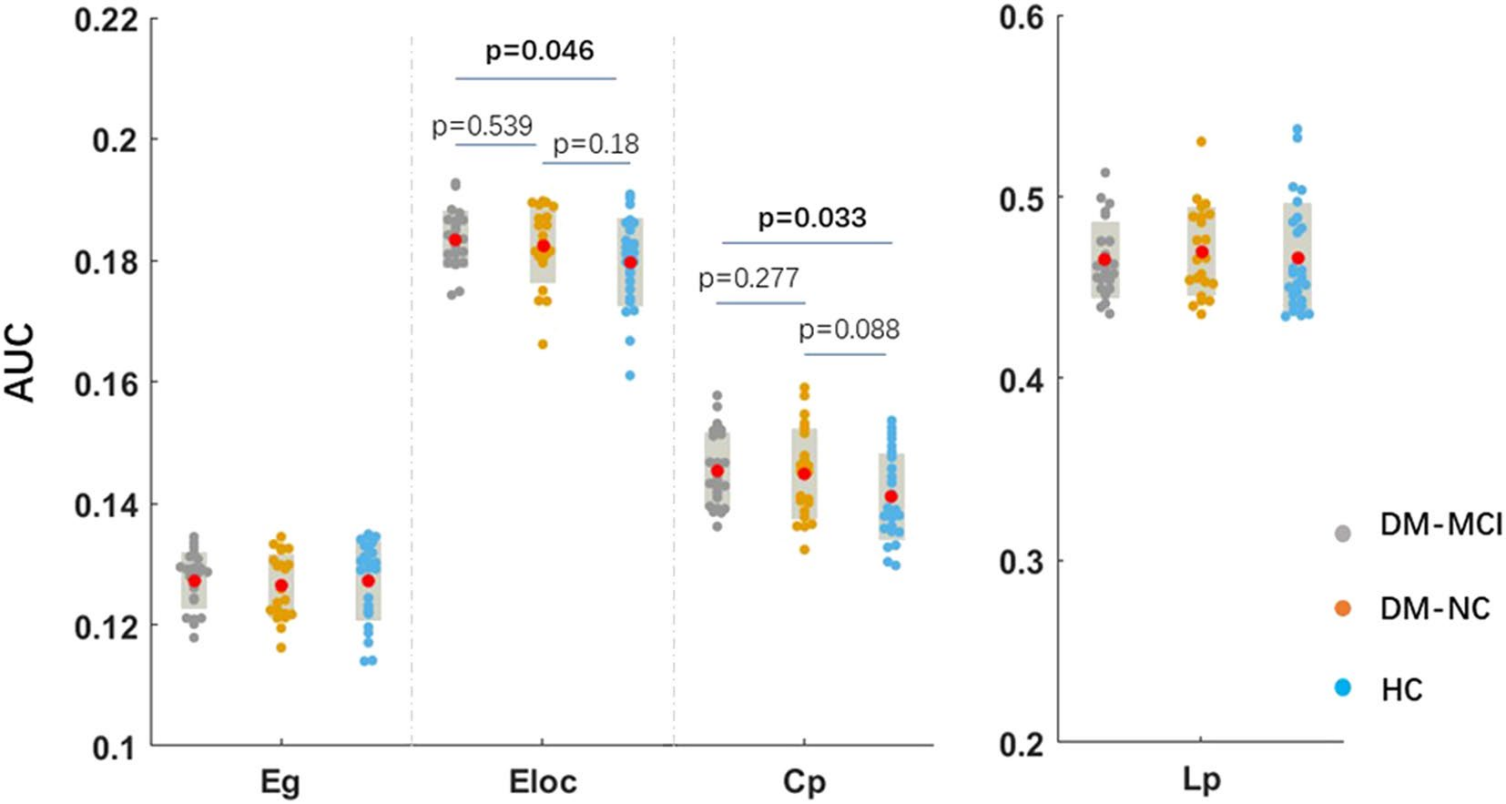
Global measurements of functional network analysis in the three groups. Graphs showing the Eg, Eloc, Cp and Lp values for DM-MCI (gray), DM-NC (orange) and healthy controls (blue). The red solid dots denote mean values in each group. Only T2DM-MCI group exhibited significant altered Eloc and Cp compared to healthy controls. *Eg* global efficiency, *Eloc* local efficiency, *Cp* clustering coefficient, *Lp* characteristic path length.

**Figure 5:**
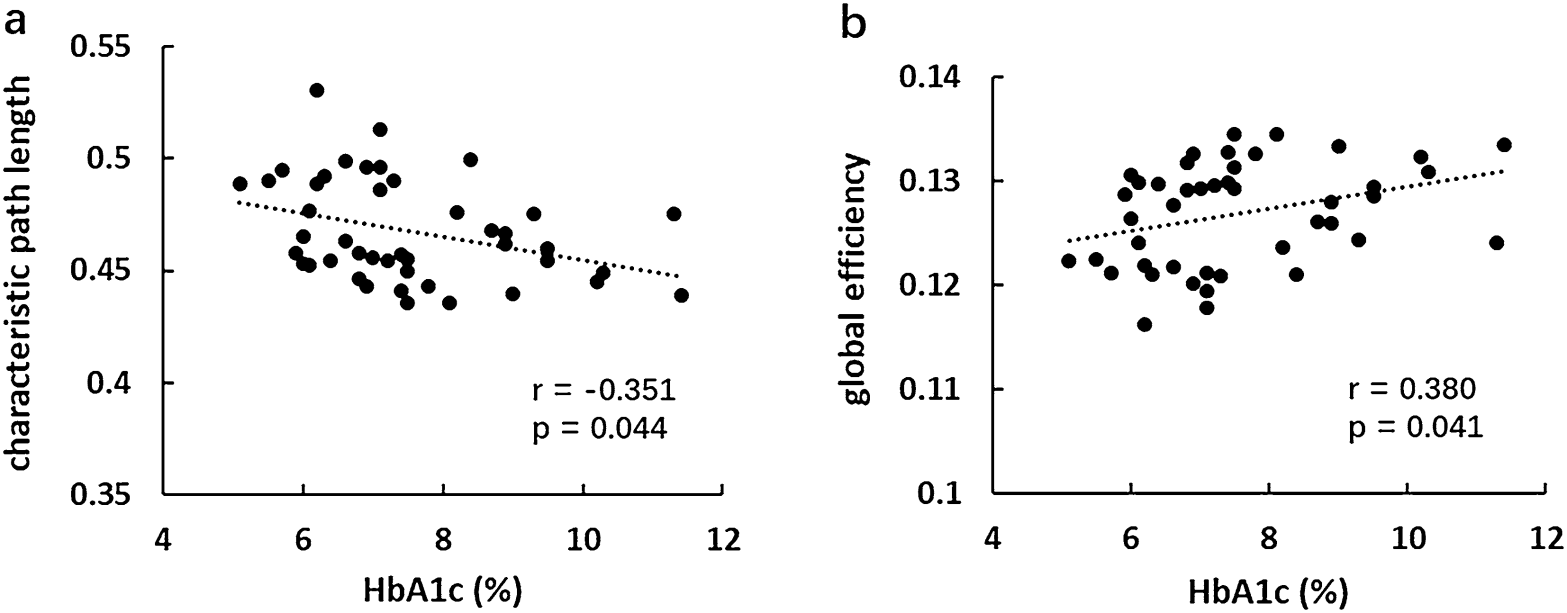
Correlations between global network properties and HbA1c: (**a**) negative correlation between Lp and HbA1c levels. (**b**) positive correlation between Eglob and HbA1c levels in T2DM patients (verticle axis: AUC).

All the three groups exhibited economical small-world organization (σ = γ/λ and σ > 1, respectively)^19^, that combined the topological advantages of both regular network and random network. There were no intergroup differences in global efficiency and characteristic path length among the three groups. The DM-MCI group exhibited a significant elevated local efficiency and clustering coefficient compared to the HCs, while the intergroup difference between the DM-NC group and HCs was not significant (Fig. 4). Furthermore, in all the T2DM patients, Lp showed a weakly negative correlation with HbA1c (r = − 0.351, *p* = 0.044), whereas Eg showed a positive correlation (r = 0.380, *p* = 0.041; Fig. 5). Correlations between any other global network properties and clinical measurements were not statistically significant (*p* > 0.05).

### Altered regional nodal characteristics

**Figure 6:**
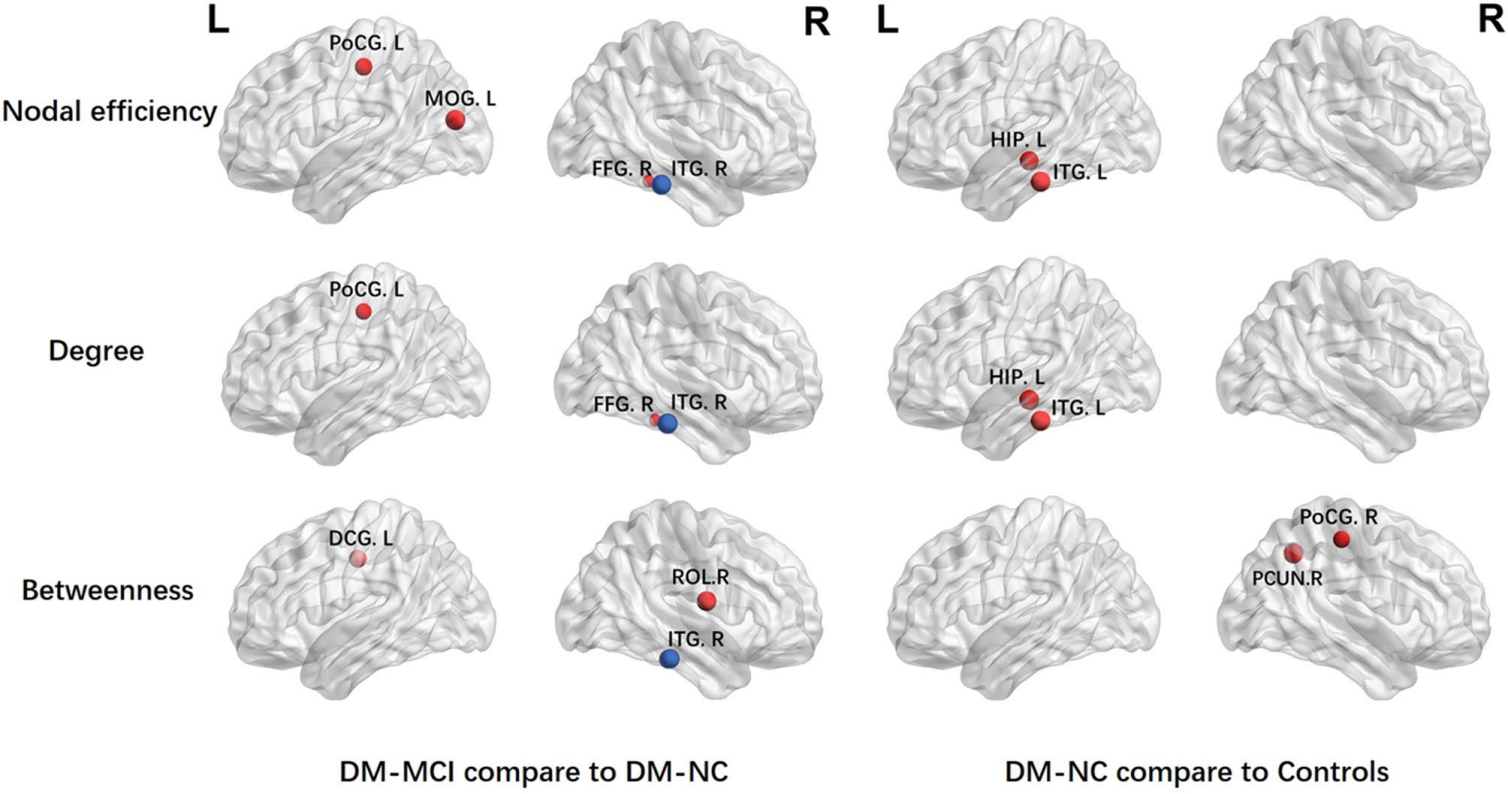
Brain regions with significantly altered network properties of nodal efficiency, nodal degree and nodal betweenness centrality among T2DM patients and healthy controls. Red: increased nodal characteristics in DM-NC or DM-MCI groups. Blue: decreased nodal characteristics in DM-MCI group. The diameter of the ball donates the F-values. *L* left, *R* right, *PoCG* Postcentral gyrus, *MOG* Middle occipital gyrus, *HIP* Hippocampus, *ITG* Inferior temporal gyrus, *FFG* Fusiform gyrus, *DCG* Median cingulate and paracingulate gyri, *PCUN* Precuneus, *ROL* Rolandic operculum.

The results of brain regions showing significant between-group differences (*p* < 0.05) among DM-MCI, DM-NC and HCs in at least one of the three nodal characteristics (efficiency, degree and betweenness) are summarized in Table 3. Compared to the HCs, the DM-NC group showed only increased nodal characteristics in the right postcentral gyrus, precuneus, left hippocampus and inferior temporal gyrus. No decreased nodal characteristics were detected. Furthermore, the DM-MCI group showed increased nodal characteristics in the left median cingulate and paracingulate gyri, middle occipital gyrus, postcentral gyrus and the right fusiform gyrus, but decreased nodal characteristics in the right inferior temporal gyrus, compared to the DM-NC group. Figure 6 shows the nodal efficiency, degree and betweenness centrality alternations among the three groups.

**Table 3:** Regions with altered nodal characteristics in type 2 diabetes patients.

AAL No. and brain regions	Abbreviation	*p* values		
		Nodal efficiency	Degree	Betweenness
**Increased in DM-NC than HC**				
37 Hippocampus_L	HIP.L	**0.046**	**0.044**	0.063
58 Postcentral gyrus_R	PoCG.R	0.131	0.118	**0.020**
68 Precuneus_R	PCUN.R	0.337	0.269	**0.036**
89 Inferior temporal gyrus_L	ITG.L	**0.035**	**0.030**	0.081
**Increased in DM-MCI than DM-NC**				
18 Rolandic operculum_R	ROL.R	0.662	0.906	**0.048**
33 Median cingulate and paracingulate gyri_L	DCG.L	0.053	0.052	**0.047**
51 Middle occipital gyrus_L	MOG.L	**0.038**	0.050	0.084
56 Fusiform gyrus_R	FFG.R	**0.046**	**0.044**	0.164
57 Postcentral gyrus_L	PoCG.L	**0.026**	**0.034**	0.456
**Decreased in DM-MCI than DM-NC**				
90 Inferior temporal gyrus_R	ITG.R	**0.010**	**0.008**	**0.019**

Corrected *p* < 0.05 are shown in bold.

AAL: automated anatomical labeling.

## Discussion

This study is, to our knowledge, the first to apply ReHo and network-connectivity approaches conjunctively to study the complex functional activity at resting-state in T2DM in one dataset. The observations included: (1) altered regional synchronization in DM-MCI versus DM-NC subjects was demonstrated. The DM-MCI group exhibited significantly decreased ReHo values in the left inferior/middle occipital gyrus and the right inferior temporal gyrus, as well as increased ReHo values in the frontal gyrus, compared to the DM-NC group. (2) at a regional level, altered nodal characteristics (efficiency, degree centrality and betweenness) were also detected in the DM-MCI group (decreased in the right inferior temporal gyrus). (3) increased ReHo and nodal characteristics in several brain regions in the DM-NC group (compared to controls) may contribute some clues for the early detection of T2DM related cognitive impairment. (4) altered ReHo of some brain regions and global network properties were related to cognitive assessments and HbA1c.

Significantly decreased ReHo values, which indicate decreased neural coherence, were found in the occipital gyrus and inferior temporal gyrus only in the DM-MCI patients. This is a major finding of this study. Previous rs-fMRI studies always have focused on comparisons between general T2DM patients, including those with normal or impaired cognition, and HCs. Typically, more regions with decreased neural activity (vs. increased) were found in the majority of previous studies. For example, T2DM patients exhibited lower ReHo values in the occipital lobe, postcentral gyrus, and middle temporal gyrus in one study^15^, and in the fusiform gyrus and precentral gyrus in another study^16^. In addition, ReHo values were found to be decreased in the occipital lobe, temporal lobe, postcentral gyrus, and cerebellum in both T2DM patients with or without microangiopathy compared to HCs^31^. Similar regions demonstrated decreased ReHo values in the present study, such as the occipital and temporal gyrus. Furthermore, decreased ReHo values in the inferior temporal gyrus were found, which are regions important for visual processing and representation of complex object features, as well as the early recognition of numbers and words^32^. Decreased neural synchronization in this region may therefore contribute to patients’ poor performance on cognitive tests. More importantly, DM-NC patients only exhibited elevated ReHo in several regions; no cortical areas exhibited significantly decreased ReHo values. Therefore, we speculate that the decreased regional synchronization revealed in T2DM patients in previous studies may have mainly resulted from or have been closely related with the cognition decline.

Patients with cognitive dysfunction typically showed decreased regional synchronization. However, some studies indicated that there were a few brain regions with enhanced ReHo values in MCI or AD patients^33,34^. Increased ReHo values in the medial frontal gyrus, anterior cingulate gyrus, precuneus and insula^15,16,31^ were also found in T2DM subjects relative to controls. Our results detected higher ReHo values in the left angular and superior temporal gyrus in the DM-NC group, as well as in the frontal lobe in the DM-MCI group. These results indicated enhanced neuronal synchronization in the functional clusters or brain regions. Since a majority of subjects in the DM-MCI group (14/25) and the DM-NC group (14/25) had a diabetes duration of more than five years, we postulate that this could reflect a compensation in those areas mentioned above after long-term weakened neural activities in the temporal lobe. Recruiting more T2DM volunteers and separating them by their disease duration may potentially address this issue in future studies.

MMSE and MoCA scores were negatively correlated with the ReHo values in the right middle frontal gyrus and the superior frontal gyrus (medial orbital). Lower MMSE or MoCA scores indicated more impaired cognitive functions, which are associated with decreased information processing speed. We note that these regions were included in or very adjacent to those regions which showed increased RoHo in the DM-MCI group. As higher ReHo values in the frontal gyrus might be interpreted as a compensatory mechanism for reduced neural activities to maintain cognition function^35^, more severely impaired cognition may presumably arouse relatively enhanced ReHo in these regions. ReHo values in the cuneus were reported to be negatively correlated with the Complex Figure test and Trail-Making test in T2DM patients^15^. In the current study, a negative correlation was also found in the left cuneus between ReHo and HbA1c; this supports the idea that T2DM patients may benefit from regular blood glucose control to prevent cognitive decline. These brain activity alterations are likely a gradual process related to cognitive decline, diabetic duration and severity.

The graph theory analysis of functional brain networks revealed abnormal architecture in T2DM patients. Previous studies demonstrated higher normalized Cp and Eloc, and some also found lower characteristic path length Lp in a group of T2DM participants compared to healthy controls^19–21^. In the present study, the cognitive status was considered and the T2DM participants were subdivided into two groups. We found only DM-MCI subjects exhibited altered global network characteristics, measured as increased Cp and Eloc, as compared to controls. Cp quantifies the number of connections between the nearest neighbors of a region as a proportion of the maximum number of possible connections. The combination of a high Cp and a high Eloc reflects high local specialization of the brain in processing information, and more efficiency in synchronizing neural activity between brain regions^36^. This result seemed strange as it implying that the whole brain networks were better organized or enhanced in DM-MCI than those in healthy controls. In the research of the functional network among T2DM patients, prediabetes patients and healthy controls, similar results to our study were found. This mainly suggesting a compensation mechanism of the functional whole-brain network in the cognition decline stage. Combined with our ReHo analysis, we obtained a similar conclusion. The altered Cp and Eloc revealed in previous studies^19–21^ were more closely related with cognitive decline. The correlation analysis between Eg and Lp and clinical measurements also supported that regular blood glucose control may help to prevent cognitive decline.

Nodal efficiency, degree and betweenness reflect the extent of integration between the immediate neighbors of a given node, and quantify how much information may traverse the node^37^. As increased nodal characteristics were found in the DM-NC and DM-MCI groups in some brain regions, decreased nodal characteristics were only detected in the right inferior temporal gyrus in DM-MCI group. This result was partly in accordance with our ReHo findings (Table 2 and Fig. 2). Reduced ReHo and nodal characteristics may attribute to disrupted visual processing and memory network function, which were relevant with inferior temporal gyrus^32^, and therefore be associated with patients’ slower responses when completing cognitive tests. Interestingly, we noticed that there were more findings related with inferior temporal gyrus in T2DM patients. The decrease of functional activity of the inferior temporal gyrus is closely related to memory decline^38^, and node properties of the inferior temporal gyrus were positively correlated with BMI^21^.

The current study has some limitations. First, as rigorous clinical diagnosis of diabetes-related cognitive impairment remains a challenge, dividing the T2DM patients into subgroups can be subject to inaccuracy. Second, the small sample size in our study may have influenced our detection of areas with altered ReHo and functional brain network measurements. Third, although we detected several brain areas with altered ReHo or nodal parameters, the accurate function of some of these regions is complicated and remains unclear. Therefore, it is difficult to provide accurate explanations regarding changes in these regions. Forth, it is essential to differentiate the true signal of interest from other noise-related fluctuations in fMRI. To reduce the effect of physiological signals (respiratory and cardiac artifacts), the fMRI data were filtered by using a band pass filter (0.01–0.1 Hz)^39^ and linear regression with the averaged time series. In further studies, physiological signals should be measured during rs-fMRI acquisition and correction for physiological noise will be performed. Finally, we studied the cross-sectional differences but not the transition from normal to impaired cognition in T2DM. Further studies with longitudinal follow-up times are necessary to confirm these findings.

In conclusion, this study compared both neuronal synchronization and functional brain network characterizations in T2DM participants with cognitive impairment and those with normal cognition. The findings demonstrated significantly altered neuronal synchronization, as well as functional brain networks properties (increased Cp and Eloc, and altered nodal characteristics) in some regions, such as the right inferior temporal gyrus, that were significantly related to cognition. Moreover, our findings suggested some alteration is already apparent in the DM-NC stage prior to MCI. Thus, the application of rs-fMRI and graph theory-based network analysis may facilitate early detection and treatment of T2DM-related MCI clinically, and offers an approach to understand the neuropathological mechanisms of T2DM-related cognitive impairment.
